# Community-based Guinea worm surveillance in Chad: Evaluating a system at the intersection of human and animal disease

**DOI:** 10.1371/journal.pntd.0009285

**Published:** 2021-03-18

**Authors:** Beth L. Rubenstein, Sharon L. Roy, Karmen Unterwegner, Sarah Yerian, Adam Weiss, Hubert Zirimwabagabo, Elisabeth Chop, Mario Romero, Philip Tchindebet Ouakou, Tchonfienet Moundai, Sarah Anne J. Guagliardo

**Affiliations:** 1 Division of Parasitic Diseases and Malaria, Center for Global Health, Centers for Disease Control and Prevention, Atlanta, Georgia, United States of America; 2 Guinea Worm Eradication Program, The Carter Center, Atlanta, Georgia, United States of America; 3 Guinea Worm Eradication Program, The Carter Center, N’Djamena, Chad; 4 Guinea Worm Eradication Program, Ministry of Public Health, N’Djamena, Chad; RTI International, UNITED REPUBLIC OF TANZANIA

## Abstract

**Background:**

Guinea worm is a debilitating parasitic infection targeted for eradication. Annual human cases have dropped from approximately 3,500,000 in 1986 to 54 in 2019. Recent identification of canine cases in Chad threatens progress, and therefore detection, prevention, and containment of canine cases is a priority. We investigated associations between disease knowledge, community engagement, and canine cases in Chad to identify opportunities to improve active surveillance.

**Methods:**

We surveyed 627 respondents (villagers, local leaders, community volunteers, and supervisors) across 45 villages under active surveillance. Descriptive statistics were analyzed by respondent category. Logistic regression models were fitted to assess the effects of volunteer visit frequency on villager knowledge.

**Results:**

Knowledge increased with respondents’ associations with the Guinea worm program. Household visit frequency by community volunteers was uneven: 53.0% of villagers reported visits at least twice weekly and 21.4% of villagers reported never being visited. Villagers visited by a volunteer at least twice weekly had better knowledge of Guinea worm symptoms (OR: 1.71; 95% CI: 1.04–2.79) and could name more prevention strategies (OR: 2.04; 95% CI: 1.32–3.15) than villagers visited less frequently. The primary motivation to report was to facilitate care-seeking for people with Guinea worm. Knowledge of animal “containment” to prevent contamination of water, knowledge of rewards for reporting animal cases, and ability to name any reasons to report Guinea worm were each positively correlated with village canine case counts.

**Conclusions:**

Community volunteers play crucial roles in educating their neighbors about Guinea worm and facilitating surveillance. Additional training and more attentive management of volunteers and supervisors could increase visit frequency and further amplify their impact. Emphasizing links between animal and human cases, the importance of animal containment, and animal rewards might improve surveillance and canine case detection. The surveillance system should be evaluated routinely to expand generalizability of data and monitor changes over time.

## Author summary

Guinea worm eradication depends on detecting people and animals with the disease in order to prevent transmission. The situation in Chad is especially urgent because the number of domestic dogs with Guinea worm has been rapidly increasing in the past five years, and now represents the vast majority of Guinea worm cases globally. The global Guinea Worm Eradication Program, the Chad Guinea Worm Eradication Program, and the Chad Ministry of Public Health requested an investigation to evaluate the associations between Guinea worm knowledge, community engagement, and case detection and to investigate how the active surveillance system could be improved to more effectively detect and contain every Guinea worm case. We surveyed 627 people across 45 villages in Chad. We found that Chad’s system for monitoring Guinea worm operates unevenly, with some villagers receiving frequent visits by community volunteers and other villagers reporting never being visited. Villagers visited by a community volunteer at least twice per week had much better knowledge of Guinea worm symptoms and prevention, compared with villagers visited less frequently. Community volunteers are instrumental to Guinea worm eradication efforts in Chad because they transmit key educational messages that facilitate disease detection. More efforts should be made to increase volunteer accountability and to provide volunteers and their supervisors with targeted, high quality training.

## Introduction

Guinea worm disease (dracunculiasis) is a debilitating parasitic infection and neglected tropical disease. The infection is typically spread by consuming water containing copepods (freshwater crustaceans that usually range from 1–5 millimeters in length) that are infected with *Dracunculus medinensis* larvae. After consumption, the copepods die and the larvae are released in the body. The worms mature and mate 60–90 days post-infection and, during the next 10–14 months, the fertilized female grows up to 1 meter in length and migrates to the skin [1]. If immersed in water, the worm emerges through a blister and discharges its larvae, which are then consumed by copepods, starting the cycle again. The emergence of adult female Guinea worms from the skin can be painful and slow. A person with Guinea worm disease is unable to work for an average of 8.5 weeks, causing a significant financial and social burden for affected communities [2].

The global Guinea Worm Eradication Program (GWEP) was initiated by the United States Centers for Disease Control and Prevention (CDC) in 1980, and since 1986, ministries of health from affected countries, The Carter Center, and the World Health Organization have led eradication efforts. Since the launch of the GWEP, the number of human cases reported annually have dropped from approximately 3,500,000 across 20 countries in 1986 to 54 cases across three countries in 2019, 49 of which occurred in The Republic of Chad [3,4].

Guinea worm in domestic dogs in Chad was identified in 2012, and canine cases have rapidly increased ever since [3]. In 2019, Chad reported 1,935 canine cases and 47 feline cases, representing 99% of reported animal infections globally [3]. These patterns suggest that animal hosts, particularly domestic dogs, are sustaining parasite transmission in Chad—previous investigations have shown that dogs and humans are infected by the same *D*. *medinensis* parasites [5]. Historical accounts describe canine infections in South and Central Asia [6–12], but dog infections decreased over time with the decline in human infections [1,13]. As with humans, it is believed that dogs may be infected through consumption of contaminated water [14–16]. In addition, investigations in Chad point to canine consumption of raw fish and frogs as a possible cause of infections since they can serve as transport or paratenic hosts [15,17–20].

The situation with dogs in Chad presents a challenge to Guinea worm surveillance and, by extension, to eradication. Within the Chad Guinea Worm Eradication Program (CGWEP), surveillance and eradication are closely linked because the objective of the surveillance system is to detect and “contain” every case of Guinea worm in Chad, meaning that human and canine cases are kept away from water sources to prevent contamination and future spread. Containment is operationalized by tethering infected dogs until all worms are removed and wounds are fully healed, and by identifying human cases within 24 hours of worm emergence and providing medical care at a Guinea worm containment center, medical clinic, or hospital until all worms are removed [21]. Timing is critical for containment; worm emergence may occur within a few hours of development of a visible blister, and the human case or infected animal can quickly contaminate water sources. Thus, surveillance is part of the intervention because cases that are detected early can be contained.

CGWEP is part of the Chad Ministry of Public Health (MOPH) and supports active, community-based surveillance in 2,147 villages with endemic transmission or high-risk of imported cases [22]. The remaining, non-endemic areas of the country are covered by a passive surveillance system. Each active surveillance site has at least two volunteers who are responsible for conducting daily household searches for possible cases of disease in humans and animals. Additionally, volunteers provide health education to villagers about Guinea worm disease biology and transmission, signs and symptoms, disease prevention methods, case reporting, and cash rewards for human and animal cases. The CGWEP uses disease awareness and cash rewards to incentivize villagers to report Guinea worm cases. Cash rewards vary from 5,000 to 50,000 CFA (≈8 to 80 USD), depending on the type of case (animal or human) and timeliness of report (before or after worm emergence) [22]. Volunteers report possible cases to their supervisors. Volunteers are not paid, but they receive incentives such as t-shirts, soap, training, and opportunities to earn cash rewards when they identify confirmed cases of Guinea worm. Supervisors are paid program staff, and typically manage 16–40 volunteers distributed across 8–15 villages.

The CGWEP has been adapted in recent years to better identify canine cases through expanded veterinary/animal health training courses, and more refined rewards and reporting requirements [22]. However, no formal assessment of the surveillance system had been conducted. Our evaluation sought to answer two questions about active surveillance for Guinea worm disease in Chad: 1) What is the relationship between Guinea worm knowledge, community engagement, and canine case detection? and, 2) How might the surveillance system be improved to more effectively detect and contain every Guinea worm case in the endemic area of Chad? The evaluation focused specifically on active surveillance sites because active sites are where the majority of CGWEP activity is concentrated.

## Methods

### Ethics statement

Data collected for this evaluation were considered part of routine Guinea worm surveillance activities by the Chad Ministry of Public Health. This project was given a non-research determination by the delegated authority at the Center for Global Health, Centers for Disease Control and Prevention (protocol #: 0900f3eb819fb155).

A survey was carried out in Chad from September 11–28, 2019 among a sample of villagers, village leaders, CGWEP volunteers, and supervisors working in active surveillance sites.

#### Site selection

**Fig 1:**
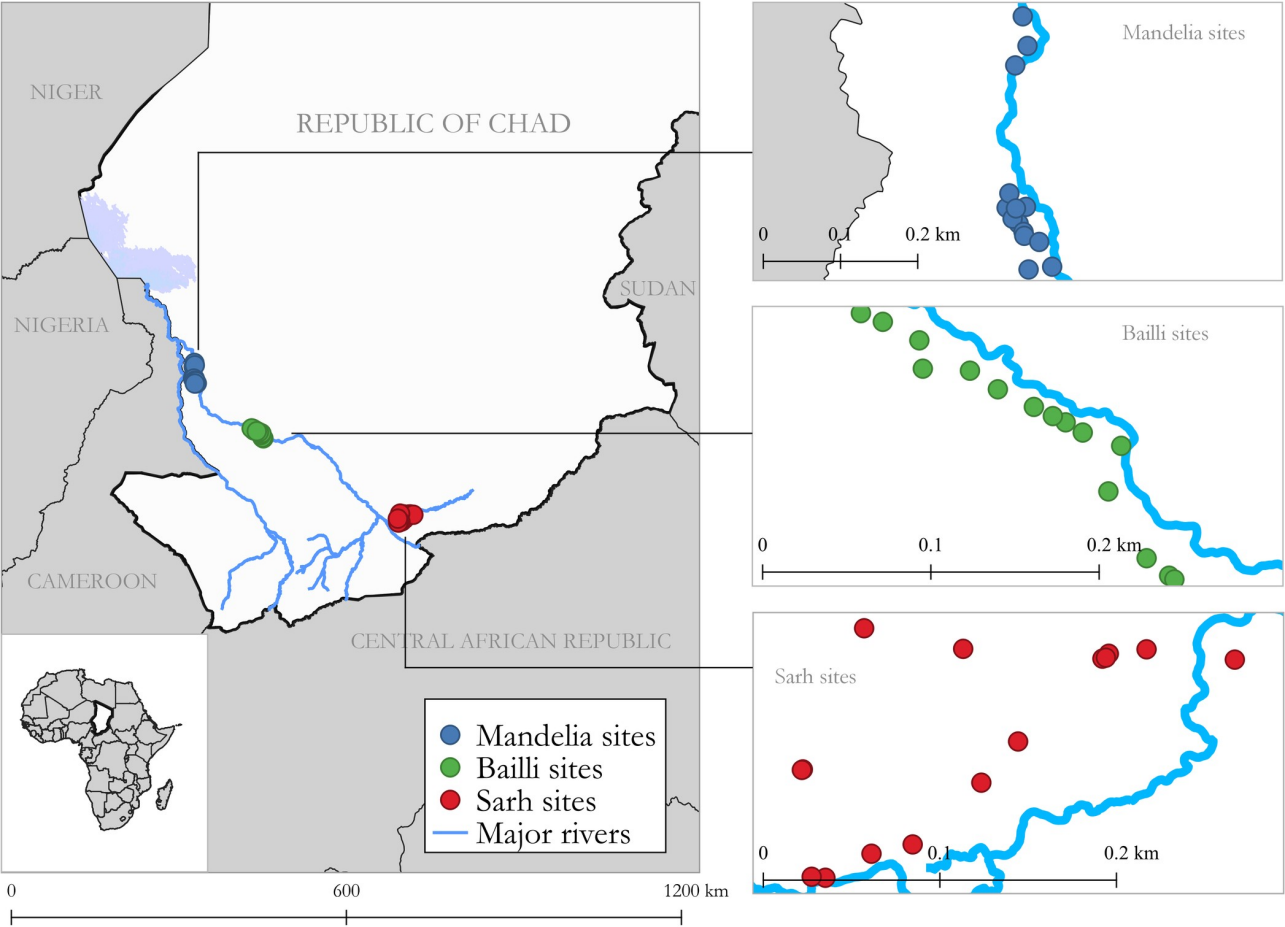
Map of southern Chad showing the locations of 45 villages purposefully selected for participation in the survey to evaluate the Chad Guinea Worm Eradication Program active surveillance system, September 2019.

Forty-five villages under active surveillance were selected for participation in the survey (Fig 1). The number of villages was chosen based on the amount of time and effort that the program could reasonably devote to this evaluation. We selected three geographic areas of study, based on the cumulative number of canine cases collected through surveillance data from 2012 to 2019. We included the Sarh area in southeast Chari because it is a known Guinea worm hotspot, with 398 cumulative canine cases distributed across 51 villages from 2012 to 2019 [17,22]. The Bailli area was also included because of its high level of canine cases (404 cumulative cases across 39 villages), and because of the increase in canine cases observed in recent years (298 canine cases in 2019, compared with 129 in 2018). The Mandelia area in northwest Chari was selected because of its moderate level of canine cases (177 cumulative canine cases distributed across 21 villages).

Fifteen villages from each geographic area were selected, including five villages with high canine case counts chosen purposively and 10 villages selected at random. “Villages with high canine case counts” were defined as villages with at least 15 canine cases during the period 2012 to 2018 (range: 15–193 cases). When more than five villages within a geographic area had high canine case counts, sites were purposively selected on the basis of accessibility. Randomly selected villages were chosen based on proximity to the purposively selected villages (i.e., within a 5–8 km buffer from the purposively selected villages, with buffer size dependent on the density of villages in the surrounding area). We excluded villages that were inaccessible by vehicle during the rainy season when data were collected.

#### Respondent selection

Within villages, data collectors recruited four categories of respondents: villagers, village leaders, CGWEP volunteers, and supervisors. Participation was optional for all respondents. We identified villagers by selecting households using a modified version of the random walk method [23]. First, data collectors identified three to four geographically distributed landmarks in the village (e.g., a school, a tree, a person’s house). Next, they randomly selected one landmark using either a phone application or a piece of paper drawn from a hat. On arrival at the selected landmark, if there were multiple paths, the data collectors determined which direction to travel by spinning a bottle on the ground. Then, they walked in a straight line visiting all households in their path until they surveyed 10 individuals from different households. At each household, the self-identified head of household was invited to participate. If the head of the household was unavailable, data collectors invited another adult to participate in the survey. Participation was limited to one individual per household.

Village leaders were always invited to participate in the survey. Two CGWEP volunteers in each village were also invited to participate. In villages with more than two volunteers, we randomly selected two individuals from the CGWEP roster of all volunteers in that location. In each village, every supervisor responsible for overseeing activities was selected.

#### Questionnaires

Separate questionnaires were developed for villagers (with additional questions for village leaders), volunteers, and supervisors. The villager/village leader questionnaire included demographic questions (age, sex, education, occupation, and ownership of dogs and cats), frequency of volunteer visits, sources of Guinea worm information, knowledge of Guinea worm, knowledge of the CGWEP reward system for reporting cases, and reasons for reporting cases. At the beginning of the survey, villagers and village leaders were asked to identify Guinea worm from two photographs (one of a person with an emerging worm and one of a dog with an emerging worm). The questionnaires for volunteers and supervisors included similar questions on demographics and Guinea worm knowledge, as well as household visit frequency and reporting. In addition, the volunteer and supervisor questionnaires gathered information about professional roles and responsibilities, experience and training, and availability of equipment and supplies. For all the questionnaires, most of the questions about Guinea worm knowledge and reporting were open-ended and allowed multiple answers (see Table 1).

**Table 1:** Descriptive statistics by respondent category for the survey to evaluate the Chad Guinea Worm Eradication Program active surveillance system: September 2019.

	Respondent Category			
	Villagers	Village Leaders	Volunteers	Supervisors^§^
	N = 468	N = 48	N = 85	N = 26
Female, *n* (%)	251 (53.6)	1 (2.1)	46 (54.1)	4 (15.4)
Age in years, mean (SD)*	36.0 (15.0)	57.0 (14.7)	34.5 (11.3)	32.8 (8.0)
Completion of primary school or higher, *n* (%)	81 (17.3)	8 (16.7)	25 (29.4)	26 (100)
Completion of secondary school or higher, *n* (%)	17 (3.6)	1 (2.1)	3 (3.5)	11 (42.3)
Any post-secondary education, *n* (%)	6 (1.3)	1 (2.1)	0 (0.0)	7 (26.9)
Farmer, *n* (%)	345 (73.7)	44 (91.7)	72 (84.7)	---
Dog owners, *n* (%)	186 (39.7)	21 (43.8)	---	---
Cat owners, *n* (%)	133 (28.4)	10 (20.8)	---	---
Frequency of volunteer visits				
Every day, *n* (%)	22 (4.7)	8 (16.7)	22 (25.9)	---
4–6 times per week, *n* (%)	40 (8.6)	3 (6.3)	18 (21.2)	---
2–3 times per week, *n* (%)	186 (39.7)	21 (43.8)	39 (45.9)	---
1 time per week, *n* (%)	52 (11.1)	3 (6.3)	3 (3.5)	---
Less often than weekly, *n* (%)	20 (4.3)	2 (4.2)	3 (3.5)	---
Never, *n* (%)	100 (21.4)	8 (16.7)	0 (0.0)	---
Unknown, *n* (%)	48 (10.3)	3 (6.3)	0 (0.0)	---
Sources of Guinea worm information				
Village volunteer, *n* (%)	308 (65.8)	24 (50.0)	---	---
Health facility, *n* (%)	54 (11.5)	6 (12.5)	---	---
CGWEP staff, *n* (%)	299 (63.9)	42 (87.5)	---	---
Village chief, *n* (%)	5 (1.1)	---	---	---
Teacher/school, *n* (%)	5 (1.1)	0 (0.0)	---	---
Radio/television, *n* (%)	21 (4.5)	2 (4.2)	---	---
Theater, *n* (%)	0 (0.0)	0 (0.0)	---	---
Mosque/church, *n* (%)	1 (0.2)	0 (0.0)	---	---
Market day, *n* (%)	6 (1.3)	1 (2.1)	---	---
Town crier, *n* (%)	3 (0.6)	0 (0.0)	---	---
Poster, *n* (%)	8 (1.7)	0 (0.0)	---	---
Can identify photo of Guinea worm, *n* (%)	452 (96.6)	45 (93.8)	83 (97.7)	---
Guinea worm symptoms named^⍏^				
Itching, *n* (%)	246 (52.6)	31 (64.6)	83 (97.7)	26 (100)
Burning, *n* (%)	63 (13.5)	16 (33.3)	55 (64.7)	26 (100)
Pain, *n* (%)	62 (13.3)	13 (27.1)	58 (68.2)	25 (96.2)
Swelling, *n* (%)	175 (37.4)	28 (58.3)	71 (83.5)	26 (100)
Blister, *n* (%)	188 (40.2)	31 (64.6)	66 (77.7)	24 (92.3)
Wound, *n* (%)	230 (49.2)	27 (56.3)	74 (87.1)	26 (100)
Emerging worm, *n* (%)	149 (31.8)	15 (31.3)	37 (43.5)	3 (11.5)
Total number of symptoms identified, mean (SD)*	2.38 (1.6)	3.35 (1.5)	5.22 (1.5)	6.00 (0.4)
Zero symptoms named, *n* (%)	81 (17.3)	1 (2.1)	1 (1.2)	0 (0.0)
Reasons named for reporting Guinea worm (motivation)^⍏^				
To receive a reward, *n* (%)	100 (21.4)	9 (18.8)	11 (12.9)	10 (38.5)
To get care, *n* (%)	237 (50.6)	21 (43.8)	30 (35.3)	3 (11.5)
To protect the community, *n* (%)	33 (7.1)	2 (4.2)	14 (16.5)	3 (11.5)
For the health of the community, *n* (%)	43 (9.2)	8 (16.7)	20 (23.5)	26 (100)
To stop the transmission of Guinea worm, *n* (%)	38 (8.1)	9 (18.8)	23 (27.1)	9 (34.6)
To eradicate Guinea worm, *n* (%)	20 (4.3)	6 (12.5)	15 (17.7)	8 (30.8)
Total number of reasons for reporting identified, mean (SD)	1.01 (0.7)	1.15 (0.7)	1.33 (0.8)	1.27 (0.5)
Zero reasons named, *n* (%)	111 (23.7)	7 (14.6)	7 (8.3)	0 (0.0)
Reward system, knowledge of ____:				
Presence of reward for reporting human cases of Guinea worm, *n* (%)	399 (85.3)	47 (97.9)	81 (96.4)	---
Amount for infected patient self-reporting Guinea worm, *n* (%)	209 (44.7)	34 (70.8)	78 (91.8)	26 (100)
Amount if someone other than the infected patient reports Guinea worm, *n* (%)	182 (38.9)	32 (66.7)	78 (91.8)	26 (100)
Presence of reward for reporting cases of Guinea worm in dogs or cats, *n* (%)	398 (85.0)	42 (87.5)	83 (98.8)	---
Reward system, knowledge of ____:				
The requirement for infected dogs or cats to be tethered until the wounds are fully healed to earn a Guinea worm reward, *n* (%)	268 (57.3)	29 (60.4)	66 (77.7)	24 (92.3)
Amount if the owner of a dog or cat reports that their animal has a blister or swelling *before* a Guinea worm emerges, *n* (%)	193 (41.2)	34 (70.8)	63 (74.1)	25 (96.2)
Amount if someone other than the owner of a dog or cat reports an animal with a blister or swelling *before* a Guinea worm emerges, *n* (%)	129 (27.6)	28 (58.3)	60 (70.6)	24 (92.3)
Amount if the owner of a dog or cat reports an animal with a wound or an emerging Guinea worm, *n* (%)	50 (10.7)	13 (27.1)	51 (60.0)	25 (96.2)
Amount if someone other than the owner of a dog or cat reports an animal with a wound or an emerging Guinea worm, *n* (%)	38 (8.1)	9 (18.8)	43 (50.6)	26 (100)
Amount if owner of a dog or cat reports any signs and symptoms of Guinea worm and no worm emerges, *n* (%)	64 (13.7)	13 (27.1)	45 (52.9)	26 (100)
Amount if someone other than the owner of the dog or cat reports any signs and symptoms of Guinea worm and no worm emerges, *n* (%)	98 (20.9)	19 (39.6)	59 (69.4)	25 (96.2)
Guinea worm prevention strategies named^⍏^				
Drinking safe water, *n* (%)	127 (27.1)	18 (37.5)	41 (48.2)	12 (46.2)
Filtering unsafe water, *n* (%)	123 (26.3)	14 (29.2)	61 (71.8)	20 (76.9)
Preventing patients from entering water sources, *n* (%)	17 (3.6)	3 (6.3)	9 (10.6)	6 (23.1)
Proper disposal of fish entrails, *n* (%)	237 (50.6)	24 (50.0)	51 (60.0)	20 (76.9)
Proper cooking of fish and aquatic animals, *n* (%)	178 (38.0)	14 (29.2)	56 (65.9)	22 (84.6)
Tethering infected dogs/cats to prevent them from entering water sources, *n* (%)	28 (6.0)	6 (12.5)	11 (12.9)	10 (38.5)
Total number of prevention strategies identified, mean (SD)*	1.52 (1.2)	1.65 (1.1)	2.69 (1.0)	3.46 (1.1)
Zero prevention strategies named, *n* (%)	123 (26.3)	8 (16.7)	2 (2.4)	0 (0.0)

* SD = standard deviation.

⍏ These questions were open-ended and multiple answers were permitted

§ In the pool of selected villages, several supervisors oversaw multiple villages within the sample, and thus the total number of supervisors was lower than the number of villages.

The structured questionnaires were developed in English and translated to written French. Data collectors were fluent in French and Chadian Arabic and, when necessary, orally translated the questionnaires from French to Chadian Arabic in the field. When a respondent did not speak Chadian Arabic or another language spoken by the data collector, local interpreters were recruited to assist.

The questionnaires and oral translation techniques were pilot tested prior to use. Nine interviewers (six CGWEP staff and three MOPH employees) conducted approximately 10 pilot interviews among a range of respondents (villagers, village leaders, volunteers, and supervisors), to allow each interviewer to practice and to refine the tools and language as needed.

Data were collected electronically using ODK-based NEMO software, developed by The Carter Center (https://getnemo.org/).

#### Case counts

Data on the numbers of confirmed canine Guinea worm cases were retrieved from the CGWEP surveillance system. “Confirmed” canine cases of Guinea worm entail visual/physical inspection by a field supervisor, and do not usually involve laboratory confirmation [22]. The data were available by village and covered the period from January 2012 to June 2019.

#### Data management and analysis

We generated descriptive findings by respondent category and calculated frequencies for categorical variables and means and standard deviations for continuous variables (Tables 1 and S1). We also generated descriptive findings by geographic area, including the mean number of confirmed Guinea worm cases at the village-level (S2 Table).

*Villager-level models*. At the villager-level, we ran a total of seven logistic regression models to assess the effects of (1) volunteer visit frequency and (2) household dog ownership on the outcome variables: (1) ability to name Guinea worm symptoms, (2) ability to name Guinea worm prevention strategies, (3) motivation to report, and (4) (for dog ownership) volunteer visit frequency (Table 2). The outcome variables (Guinea worm symptoms, prevention strategies, and motivation to report) were classified into binary variables based on naming a specified number of items (threshold). The thresholds for each outcome were defined as two correctly identified symptoms, two correctly identified prevention strategies, and any reasons for reporting (i.e., motivation to report). Thresholds were determined by calculating the mean number of correct responses for each outcome variable. (Response options were chosen to reflect the key messages that are promoted by CGWEP, such as identifying blisters as a symptom, tethering infected dogs and cats to prevent them from entering water sources, and reporting cases to stop transmission.) Visit frequency (both an outcome and predictor) was measured for volunteers based on villager reports, and possible responses were dichotomized at two days per week so that about half of the observations fell into each category. The predictor variable, dog ownership, was measured based on whether the household owned a dog, as reported by the survey respondent.

**Table 2:** Villager-Level Models: The impact of volunteer visits and dog ownership on Guinea worm knowledge (n = 468 villagers) in the survey to evaluate the Chad Guinea Worm Eradication Program active surveillance system: September 2019. Guinea worm knowledge was assessed by symptoms named, strategies named, and reasons for reporting Guinea worm.

	Outcomes							
	> 2 GW symptoms named^⍏^		> 2 GW prevention strategies named^¶^		Any reasons for reporting GW named^§^		Visited by a volunteer > 2 times per week	
**Predictor Variables**	OR (95% CI)	AIC	OR (95% CI)	AIC	OR (95% CI)	AIC	OR (95% CI)	AIC
Visited by volunteer ≥ 2 per week*	**1.71 (1.04, 2.79)**	565.46	**2.04 (1.32, 3.15)**	637.79	1.58 (0.97, 2.61)	507.30	---	
Household dog ownership*	1.57 (0.97, 2.56)	567.86	1.52 (0.89, 2.60)	647.58	**1.64 (1.06, 2.54)**	507.17	**1.87 (1.19, 2.96)**	642.12

Bold indicates statistical significance (p<0.05).

* Adjusted for clustering by village and number of dogs with Guinea worm at the village-level, 2018–2019.

⍏ Symptoms include itching, burning, pain, swelling, blister, wound, and emerging worm.

¶ Prevention strategies include drinking safe water, filtering unsafe water, preventing patients from entering water sources, proper disposal of fish entrails, proper cooking of fish and aquatic animals, and tethering infected dogs/cats to prevent them from entering water sources.

§ Reasons for reporting include to receive a reward, to get care, to protect the community, for the health of the community, to stop the transmission of Guinea worm, and to eradicate Guinea worm.

A repeated measures term (fixed effect) was included to account for clustering by village. We also adjusted for the number of confirmed canine Guinea worm cases at the village-level between 2018 and June 2019 because we hypothesized that infection levels were a common cause of the predictors and the outcomes [24].

As a secondary analysis, we explored the number of years a village was under active surveillance as a predictor of the outcome variables listed above (ability to name Guinea worm symptoms and prevention strategies, motivation to report, and volunteer visit frequency). Active surveillance start year, from CGWEP data, was used to calculate the number of years of active surveillance per village, up to and including the year 2019. Years of active surveillance were dichotomized at the mean (five years) (S3 Table).

*Village-level models*. At the village-level, we ran negative binomial models to evaluate the effect of (1) volunteer visit coverage and (2) the proportion of villagers with Guinea worm knowledge on the count of confirmed canine Guinea worm cases January 2018—June 2019 (Table 3). Visit coverage and Guinea worm knowledge may serve as indicators of CGWEP program intensity and effectiveness and could therefore affect case detection. Volunteer visit coverage was defined as the proportion of surveyed villagers in the community who were visited by a volunteer at least twice per week. The proportion of villagers with Guinea worm knowledge was defined as the proportion of surveyed villagers in the community who correctly identified a given knowledge item in the questionnaire (e.g., the proportion who identified tethering as a prevention strategy).

**Table 3:** Village-Level Models: The effect of volunteer engagement and villager knowledge on number of confirmed canine Guinea worm cases at the village-level, January 2018–June 2019 (*n* = 45 villages) in the survey to evaluate the Chad Guinea Worm Eradication Program active surveillance system.

Predictor Variables Proportion of villagers:	Crude	Adjusted
	AMR* [95% CI],	AMR*^⍏^ [95% CI],
	p-value	p-value
Identified tethering infected dogs/cats to prevent them from entering water sources as a prevention strategy	**1.07 [1.03, 1.12], 0.001**	**1.04 [1.01, 1.07], 0.020**
Identified proper disposal of fish entrails as prevention strategy	**1.02 [1.00, 1.03], 0.022**	1.00 [0.99, 1.02], 0.551
Identified proper cooking of fish and aquatic animals as a prevention strategy	1.01 [0.99, 1.02], 0.343	1.01 [0.99, 1.02], 0.441
Aware of rewards for reporting human cases	**1.04 [1.02, 1.06], 0.0002**	1.01 [0.99, 1.04], 0.186
Aware of rewards for reporting animal cases	**1.06 [1.04, 1.08], < .0001**	**1.03 [1.01, 1.05], 0.006**
Identified receiving a reward as a reason to report	**0.98 [0.97, 0.99], 0.004**	0.99 [0.98, 1.00], 0.068
Identified getting care as a reason to report	1.01 [0.99, 1.02], 0.532	1.01 [0.99, 1.02], 0.454
Visited by a volunteer ≥ twice per week	[0.99, 1.02], 0.469	0.99 [0.97, 1.00], 0.054
Could name ≥ 2 GW symptoms	[1.00, 1.03], 0.050	1.00 [0.99, 1.01], 0.990
Could name ≥ 2 GW prevention strategies	1.01 [1.00, 1.03], 0.093	1.00 [0.98, 1.02], 0.832
Could name any reasons for reporting GW^¶^	**1.02 [1.01, 1.04], 0.001**	**1.02 [1.00, 1.04], 0.048**

* Arithmetic mean ratio of confirmed canine Guinea worm cases per village from 2018–2019.

⍏ Adjusted for village selection (purposive vs. random), geographic area, and proportion of villagers that own dogs.

All models included covariates to adjust for village selection method (purposive vs. random), geographic area, and the proportion of villagers living in households that own dogs because these variables were theorized to be confounders [24].

Data management procedures and analyses were conducted in SAS 9.4 [25]. Maps were developed in the program Quantum GIS [26].

## Results

### Descriptive findings

A total of 627 individuals from 45 villages participated in the survey (468 household respondents, 48 village leaders, 85 volunteers and 26 supervisors) (Table 1). The combined participation rate for household respondents and village leaders was 98.7%; the participation rate for volunteers and supervisors was 100%. The vast majority of household respondents, village leaders, and volunteers were farmers, and most had not completed primary school. Volunteers had higher primary school completion rates (29.4%) than household respondents (17.3%) and village leaders (16.7%). Approximately 40% of villagers and village leaders owned dogs.

Knowledge of Guinea worm symptoms, motivation for reporting, rewards for reporting, and prevention strategies steadily increased with the individual’s association with the Guinea worm program (i.e., from household respondents to village leaders to volunteers to supervisors). Correct identification of a photograph of Guinea worm was close to 100% for all categories of respondents. About 70% of household respondents and 85% of village leaders could identify two or more Guinea worm symptoms. The most common motivation cited for reporting Guinea worm was to get care (50.6% for household respondents, 43.8% for village leaders), followed by receiving a reward (21.4% and 18.8%, respectively).

Knowledge about Guinea worm was usually transmitted to lay persons through official program channels. Specifically, 63.9% of villagers learned about Guinea worm from CGWEP staff and 65.8% learned about Guinea worm from village volunteers. Other sources of information about Guinea worm, such as teachers, medical providers, and religious leaders, were rare.

Overall awareness of the existence of rewards for reporting Guinea worm in people and animals was high (Guinea worm in people: 85.3% for household respondents, 96.4% for volunteers; Guinea worm in animals: 85.0% for household respondents, 98.8% for volunteers), but knowledge of specific reward amounts for different reporting scenarios varied depending on the respondent category and the scenario. Lay person knowledge was highest with regards to the reward amount for infected people self-reporting Guinea worm (44.7% for household respondents; 70.8% for village leaders) and lowest with regards to the reward amount for animals with a wound or an already emerging Guinea worm (8.1–10.7% for household respondents and 18.8–27.1% for village leaders, depending on whether the person reporting owned the animal being reported). Volunteer knowledge of reward amounts was higher for human rewards (91.8%) than animal rewards (50.6–77.7%, depending on the reporting scenario). Supervisor knowledge ranged from 92.3–100% for various scenarios for both human and animal rewards. Only 57.3% of villagers and 60.4% of village leaders knew that dogs or cats with Guinea worm must be tethered until the wound from an emerged Guinea worm is fully healed in order to earn a monetary reward.

Knowledge of prevention strategies was uneven, with “proper disposal of fish entrails” and “proper cooking of fish and aquatic animals” the most frequently cited strategies for household respondents (50.6% and 38.0%, respectively) and village leaders (50.0% and 29.2%, respectively). Prevention strategies related to containment (“preventing patients from entering water sources” and “tethering infected dogs/cats to prevent them from entering water sources”) were the least recognized strategies across all respondent categories. Of the six prevention strategies that lay persons can engage in, volunteers were only able to name 2.7 on average, and supervisors were only able to name 3.5 on average. Villagers and village leaders named 1.5 and 1.7 on average. The most commonly named strategy by volunteers was filtering unsafe water (71.8%). Containment of humans (10.6%) and animals (12.9%) were the least cited prevention strategies by volunteers. Similarly, containment of humans (23.1%) and animals (38.5%) were the least cited preventions strategies by supervisors.

Villagers were asked how frequently they were visited by community volunteers. Similarly, community volunteers were asked how frequently they visited households. The frequency of household visits by community volunteers was inconsistent, with 53.0% of villagers reporting visits at least twice a week and 21.4% of villagers reporting never being visited. In contrast, 93.0% of community volunteers reported conducting household visits at least twice a week (including 25.9% who reported daily visits), and no volunteers reported never conducting household visits. (However, visit frequency as reported by villagers and volunteers are not directly comparable because villager reports reflect visitation of a specific household, whereas volunteer reports reflect general visitation to any households in the volunteer’s catchment area.)

The availability of equipment and supplies that volunteers and supervisors had on hand at the time of the survey varied greatly, depending on the item (S1 Table). In general, both volunteers and supervisors were lacking some program forms and health education materials. For example, only about half of the volunteers (51.8%) had Guinea worm ID cards used for health education and very few volunteers and supervisors had pipe filters (8.2% for volunteers, 3.9% for supervisors) or filter cloths (2.4% for volunteers, 23.1% for supervisors). Supervisors were also lacking some supplies to manage cases: only 15.4% had tubes and 65.4% had alcohol to collect worm specimens. Although only 63.5% of volunteers were able to produce their village registers used to record program activities, this proportion is higher than we might expect, since two volunteers were interviewed per village and the volunteers share a single register. At the village level, a register was observed in 83.3% of the villages that were visited.

When analyzed by geographic area, the demographic profiles of household respondents were similar across areas, although Sarh had a significantly higher proportion of dog owners. The number of confirmed canine Guinea worm cases were similar across the three areas, as was the average number of years that the sampled villages had been under active surveillance. Several measures of knowledge varied significantly by geography, with villagers in Sarh generally having the highest level of knowledge (S2 Table).

### Villager-level models

Logistic regression models adjusted for number of village-level canine Guinea worm cases showed that villagers who were visited by a volunteer at least twice per week were more knowledgeable in comparison with villagers who were visited less frequently. Specifically, villagers who were visited at least twice per week were 1.71 times more likely to name two or more Guinea worm symptoms (95% CI: 1.04–2.79) and 2.04 times more likely to name two or more strategies for Guinea worm prevention (95% CI: 1.32–3.15) (Table 2). The crude results are not reported but were similar to the adjusted results.

After adjusting for number of canine Guinea worm cases, dog owners were more likely to name any reasons for reporting Guinea worm (OR = 1.64, 95% CI: 1.06–2.54) and were more likely to be visited by volunteers at least twice per week (OR = 1.87, 95% CI: 1.19–2.96) (Table 2). Again, the crude results were similar to the adjusted results.

Villagers residing in communities that were under active surveillance for five or more years were significantly less likely to name two or more Guinea worm symptoms (OR = 0.39, 95% CI: 0.23–0.65), and were also less likely to be visited by a volunteer at least twice per week (OR = 0.55, 95% CI: 0.35–0.87), after adjusting for the number of canine cases (S3 Table).

### Village-level models

Adjusted negative binomial regression models showed that the number of canine Guinea worm cases at the village-level increased by 4% for each percentage point increase in the proportion of villagers who identified tethering infected dogs/cats as a prevention strategy (Arithmetic Mean Ratio/AMR = 1.04, 95% CI: 1.01–1.07). Further, the number of canine cases increased by 3% for each percentage point increase in the proportion of villagers who were aware of rewards for reporting animal cases (AMR = 1.03, 95% CI: 1.01–1.05) and by 2% for each percentage point increase in the proportion who could name any reasons for reporting Guinea worm (AMR = 1.02, 95% CI: 1.00–1.04) (Table 3).

## Discussion

This was the first quantitative assessment of Guinea worm knowledge and community engagement with the CGWEP active surveillance system in Chad. This evaluation benefited from collecting data from a diverse group of respondents across multiple geographic areas in Chad and from a high response rate. The evaluation found that volunteers play a crucial role in disseminating information about Guinea worm and engaging the larger community in surveillance. The frequency of volunteer visits was a strong predictor of household knowledge about Guinea worm symptoms and prevention strategies.

The evaluation also revealed substantial variability in volunteer visit frequency, and villager and volunteer knowledge. The fact that more than one in five villagers reported never being visited by a volunteer raises concerns about the adequacy of volunteer supervision and management. Discussions with CGWEP leadership indicated that gaps in volunteer visit frequency may be partially explained by inconsistent messaging and expectations from program staff on the ground. In addition, villagers living in households that owned dogs were more likely to be visited than villagers living in households that did not own dogs. This pattern suggests volunteers target their visits to households they perceive are more likely to identify cases. Even though this is not aligned with official CGWEP guidance to visit every household every day, there is evidence that some dogs are repeatedly infected with Guinea worm and history of infection is correlated with higher worm burden [22,27]. Still, CGWEP’s rationale for including households without dogs in active surveillance activities is threefold: the risk for human transmission still exists, these households may own cats, and household members may observe other animals with Guinea worm disease inside the village, out in the fields, or while hunting. Data on the relative contribution of households without dogs to case detection may help CGWEP refine their rationale and guidance on visit frequency. Visit frequency also decreased in villages that had been under active surveillance for five or more years, suggesting program fatigue may develop over time. This is consistent with anecdotal reports of program fatigue from field staff.

With regards to variability in villager knowledge, geographic variations were notable, with villagers in Sarh generally having the highest level of knowledge. Variations could not be explained by geographic differences in dog ownership or years of active surveillance. Potential qualitative explanations for the variability in knowledge by geographic area might include accessibility issues and communication challenges associated with different languages and cultures. However, without further data, these explanations cannot be confirmed.

Gaps in villager and volunteer knowledge about prevention strategies were also concerning, especially the fact that knowledge about proper disposal of fish entrails and proper cooking of fish and aquatic animals eclipsed knowledge about containment. Containment was the least frequently cited prevention strategy by volunteers and supervisors, who are responsible for imparting this knowledge to the public. The successful certification of transmission interruption in 16 formerly endemic countries using containment and other established Guinea worm interventions provides an evidence base that is much stronger than the evidence base supporting newer prevention messages such as burying fish entrails, but our survey suggests this hierarchy of interventions has not been successfully communicated during training and education [21,28]. Similarly, approximately 40% of villagers and village leaders and 22% of volunteers did not know that infected dogs and cats must be tethered until the wounds are fully healed to earn a Guinea worm reward. This lack of knowledge could compromise containment effectiveness and significantly impact eradication efforts. Surprisingly, when asked about motivation for reporting Guinea worm, villagers and village leaders cited the provision of health care for people with Guinea worm (50.6% and 43.8%, respectively) at least twice as often as they cited receiving a reward (21.4% and 18.8%, respectively). However, awareness of a reward for reporting human and animal cases ranged from 85.0% to 97.9% among villagers and village leaders, respectively, indicating that, on this point, diffusion of health information into the communities was successful.

In addition, our analyses identified three village-level knowledge indicators that were positively associated with the number of confirmed canine cases after adjusting for confounding: 1) awareness of rewards for reporting animal cases, 2) proportion of households that identified the containment strategy of tethering infected dogs and cats to prevent them from entering water sources, and 3) ability to name any reasons for reporting Guinea worm. As awareness of rewards, containment and reasons for reporting increased, so did the number of confirmed canine cases for those villages, suggesting these elements may deserve special emphasis during training. Still, these findings could also be examples of reverse causality, whereby more canine cases lead to higher awareness about rewards, containment, and reasons for reporting. If CGWEP protocols are followed, villagers living in places with more cases will be more frequently exposed to both reward and containment procedures and general Guinea worm education. Notably, none of the other village-level indicators of volunteer engagement and Guinea worm knowledge that were analyzed predicted the number of confirmed canine cases after adjusting for confounding. This suggests that the number of canine cases is also driven by unmeasured factors, such as local epidemiologic and environmental conditions. This is consistent with findings from new research [29].

There are several limitations to this evaluation. First, the sample was not representative of the entirety of CGWEP’s activities across Chad and may be biased towards higher performing sites. This is because the survey excluded villages that were difficult for the survey teams to access during the rainy season, which is the time of year when this evaluation took place. During this time of year, poor road conditions routinely challenge supervision, training, education, and reporting. Second, there were also differences between purposively selected sites with high canine case counts, compared with randomly selected sites, including more frequent volunteer visits in the purposively selected sites. Moreover, it is important to acknowledge that the randomly selected sites are not truly random since the selection process was tied to the purposively selected sites. Third, the sample sizes for respondent categories other than villagers were small and therefore it was not possible to estimate measures of association using data from village leaders, volunteers, or supervisors. Fourth, this was a cross-sectional survey with data collected at a single point in time. Thus, reverse causality cannot be ruled out, as discussed in the village-level models of canine case counts. Fifth, visit frequency was measured based on self-report and is subject to recall errors. There are no independently verifiable program records which capture visit frequency. For household assessment of volunteer visit frequency, it is possible that the respondent who participated in the survey was not at home or otherwise unaware of a given visit. For volunteer assessment of supervisor visit frequency, volunteers may be hesitant to report infrequent supervisor visits for fear of jeopardizing their position. Finally, multiple aspects of the evaluation were dependent on accurate translation from English to French, Chadian Arabic and other languages, and the original meanings of some questions and answers may have been somewhat modified during field translations.

Our findings suggest several areas for improvement. CGWEP might consider reaffirming the centrality of volunteers and supervisors in the current system and increase the quality and frequency of their training, rather than expanding surveillance to include teachers, medical providers, religious leaders, and other stakeholders. Volunteers and supervisors are already active in their communities and, with additional training to address knowledge gaps, their impact could be further amplified. In particular, volunteer and supervisor training regarding prevention strategies should be strengthened. Containment as a cornerstone of prevention should be emphasized during training activities. While proper disposal of fish entrails and proper cooking of fish and aquatic animals are important prevention strategies, both strategies were introduced by CGWEP in recent years, and the novelty of these strategies may be competing with long-standing evidence-based strategies such as containment. More emphasis on the importance of tethering dogs and cats as a condition of earning rewards should also be considered. In addition, the program would benefit from more rigorous supervision of volunteers to improve their accountability and encourage uniform household visitation, consistent with CGWEP policies. Relatedly, the expectation of daily household visits can be communicated more clearly and emphatically. Special attention is needed in villages that have had active surveillance programs for five or more years to address fatigue and waning participation. The reward system is another critical feature of the surveillance system that warrants further investigation. At the village-level, awareness of rewards was associated with more Guinea worm cases, suggesting rewards may be a particularly important element to promote in training, but few individuals cited rewards as a motivation to report, revealing a puzzling disconnect. It is possible that villagers do not value the rewards in the manner that CGWEP has assumed and this should be explored prior to rolling out future trainings about rewards.

In the meantime, it may be beneficial to build on villagers’ and volunteers’ reported motivation to seek care and to emphasize such in staff trainings and community health education sessions. Although few people in Chad need to seek care for a Guinea worm infection, emphasizing the quality of care that is provided could incentivize participation in surveillance. At the same time, it may be useful to educate volunteers and supervisors about the link between animal and human cases so that they have a greater understanding of Guinea worm transmission and have more reasons for reporting beyond care-seeking. Notably, at the village-level, knowledge of any reason for reporting was associated with more Guinea worm cases, a signal that motivation to report may lead to improved case detection.

Finally, going forward, CGWEP’s active surveillance system should consider conducting similar external evaluations on an annual basis. Each year, the survey methodology could be applied to a new set of sites to expand the generalizability of the data. Data from more sites may also improve our understanding of what is driving the observed geographic differences in knowledge. Over time, we believe these collective efforts will enable the surveillance system to achieve its stated goal of detecting and containing every human and animal case of Guinea worm in Chad. This work could also be informative to other eradication programs that rely on community-based surveillance. At minimum, another evaluation in the dry season is highly encouraged when accessibility issues are much diminished and villages isolated by seasonal rainfall can be included—these villages are much more likely to be affected by supervision, training, education, and reporting challenges identified in this surveillance evaluation.

## Supporting information

S1 Table Additional descriptive statistics by respondent category for the survey to evaluate the Chad Guinea Worm Eradication Program active surveillance system: September 2019. (DOCX)

S2 Table Descriptive statistics by geographic area for the survey to evaluate the Chad Guinea Worm Eradication Program active surveillance system: September 2019. (DOCX)

S3 Table Villager-Level Models (Supplement): The impact of years of active surveillance on Guinea worm knowledge and volunteer visit frequency (n = 468 villagers) in the survey to evaluate the Chad Guinea Worm Eradication Program active surveillance system: September 2019. (DOCX)
