# Supplementary material for: Community-based Guinea worm surveillance in Chad: Evaluating a system at the intersection of human and animal disease
Source: PLoS Negl Trop Dis. 2021 Mar 18;15(3):e0009285. doi: 10.1371/journal.pntd.0009285 (PMC8023463; doi:10.1371/journal.pntd.0009285)
Supplement: S1 Table — (DOCX) [file pntd.0009285.s001.docx]

# S1 Table, Additional descriptive statistics by respondent category for the survey to evaluate the Chad Guinea Worm Eradication Program active surveillance system: September 2019

|  | **Respondent Category** | |  |
| --- | --- | --- | --- |
| **Volunteer-Specific Questions** | **Volunteers** | **Supervisors** | **Supervisor-Specific Questions** |
|  | N=85 | N=26 |  |
| Volunteer responsibilities named^¶^ |  |  | Supervisor responsibilities named^¶^ |
| Health education, *n* (%) | 47 (55.3) | 17 (65.4) | Health education, *n* (%) |
| Keeping the village register, *n* (%) | 2 (2.4) | 4 (15.4) | Fill out the village register in each village, *n* (%) |
| Daily search for Guinea worm rumors, suspects and cases, *n* (%) | 64 (75.3) | 16 (61.5) | Detect Guinea worm rumors, suspects and cases, *n* (%) |
| Distribute and verify Guinea worm filters, *n* (%) | 3 (3.5) | 1 (3.9) | Distribute Guinea worm filters, *n* (%) |
| Report suspect Guinea worm to the supervisor, *n* (%) | 16 (18.8) | 3 (11.5) | Report suspect Guinea worm to supervisors,  *n* (%) |
| Bandage suspects, *n* (%) | 1 (1.2) | 5 (19.2) | Write monthly reports, *n* (%) |
| --- | --- | 15 (57.7) | Supervise volunteers, *n* (%) |
| --- | --- | 8 (30.8) | Supervise Abate activities, *n* (%) |
| Guinea worm treatments named...When you find someone with Guinea worm, you should: ^¶^ |  | --- | --- |
| Bandage them, *n* (%) | 6 (7.1) | --- | --- |
| Provide health education, *n* (%) | 9 (10.6) | --- | --- |
| Investigate them, *n* (%) | 5 (5.9) | --- | --- |
| Inform village leaders, *n* (%) | 0 (0.0) | --- | --- |
| Inform Supervisor (or CGWEP staff), *n* (%) | 77 (90.6) | --- | --- |
| Total number of treatments identified, mean (SD) | 1.14 (0.6) | --- | --- |
| Zero treatments named, *n* (%) | 8 (9.5) | --- | --- |
| Experience in role |  |  | Experience in role |
| <1 year, *n* (%) | 17 (20.0) | 10 (38.5) | <1 year, *n* (%) |
| 1–2 years, *n* (%) | 16 (18.8) | 6 (23.1) | 1–2 years, *n* (%) |
| >2 years, *n* (%) | 52 (61.2) | 10 (38.5) | >2 years, *n* (%) |
|  |  |  |  |

|  | **Respondent Category** | |  |
| --- | --- | --- | --- |
| **Volunteer-Specific Questions** | **Volunteers** | **Supervisors** | **Supervisor-Specific Questions** |
|  | N=85 | N=85 |  |
| Year and frequency of training |  |  | Year and frequency of training |
| This year only (2019), *n* (%) | 70 (82.4) | 22 (84.6) | This year only (2019), *n* (%) |
| Last year only (2018), *n* (%) | 4 (4.7) | 0 (0.0) | Last year only (2018), *n* (%) |
| Both this year and last year (2019 and 2018), *n* (%) | 6 (7.1) | 2 (7.7) | Both this year and last year (2019 and 2018), *n* (%) |
| More than two years ago (2017 or earlier), *n* (%) | 4 (4.7) | 1 (3.9) | More than two years ago (2017 or earlier),  *n* (%) |
| Never received training, *n* (%) | 1 (1.2) | 1 (3.9) | Never received training, *n* (%) |
| Presence of equipment and supplies |  |  | Presence of equipment and supplies |
| Human and animal Guinea worm ID cards, *n* (%)^\|\|^ | 44 (51.8) | 26 (100) | Human and animal Guinea worm ID cards, *n* (%)^\|\|^ |
| Yellow card (for Guinea worm signs & symptoms),  *n* (%)^\|\|^ | 66 (77.7) | 21 (80.8) | Yellow card (for Guinea worm signs & symptoms), *n* (%)^\|\|^ |
| Filter cloths, *n* (%) | 7 (8.2) | 1 (3.9) | Filter cloths, *n* (%) |
| Pipe filters, *n* (%) | 2 (2.4) | 6 (23.1) | Pipe filters, *n* (%) |
| Guinea worm t-shirt, *n* (%)^\|\|^ | 46 (54.1) | 2 (7.7) | Cash reward survey form, *n* (%) |
| Village register, *n* (%)^§\|\|^ | 54 (63.5) | 24 (92.3) | Monthly calendar, *n* (%)^\|\|^ |
|  |  | 25 (96.2) | Rumor forms (human), *n* (%)^\|\|^ |
|  |  | 26 (100) | Rumor forms (animal), *n* (%)^\|\|^ |
|  |  | 23 (88.5) | Case/infection forms, *n* (%)^\|\|^ |
|  |  | 11 (42.3) | Monthly tally sheets, *n* (%) |
|  |  | 23 (88.5) | Supervisor bag, *n* (% |
|  |  | 17 (65.4) | Alcohol, *n* (%)^\|\|^ |
|  |  | 4 (15.4) | Sample tubes, *n* (%)^\|\|^ |
|  |  | 23 (88.5) | Gloves, *n* (%)^\|\|^ |
|  |  | 8 (30.8) | Gauzes, *n* (%)^\|\|^ |
|  |  | 16 (61.5) | Peanut paste (baku), *n* (%) |
|  |  |  | Bike tools^\|\|^ |
|  |  | 9 (34.6) | Chains, *n* (%)^\|\|^ |
|  |  | 9 (34.6) | Padlocks, *n* (%)^\|\|^ |
|  |  | 12 (46.2) | Bike pump, *n* (%) |
|  |  | 12 (46.2) | Tire patch kit, *n* (%) |

^†^SD=standard deviation.

^¶^ These questions were open-ended and multiple answers were permitted.
§Only one register exists per village, and multiple volunteers were interviewed per village. Therefore the number presented here may underestimate the number of registers available in each village.
^||^ These items are high-priority, and all volunteers and supervisors should have them.
