# Supplementary material for: Community-based Guinea worm surveillance in Chad: Evaluating a system at the intersection of human and animal disease
Source: PLoS Negl Trop Dis. 2021 Mar 18;15(3):e0009285. doi: 10.1371/journal.pntd.0009285 (PMC8023463; doi:10.1371/journal.pntd.0009285)
Supplement: S2 Table — (DOCX) [file pntd.0009285.s002.docx]

# S2 Table, Descriptive statistics by geographic area for the survey to evaluate the Chad Guinea Worm Eradication Program active surveillance system: September 2019

|  | **Geographic Area*** | | |  |
| --- | --- | --- | --- | --- |
|  | **Bailli** | **Mandelia** | **Sarh** | **p-value** |
|  | N=153 villagers | N=160 villagers | N=155 villagers |  |
| Number of annual canine Guinea worm cases per village, 2018–2019, mean (SD) ^†^ |  |  |  |  |
| Overall | 11.7 (16.9) | 11.9 (18.8) | 12.9 (14.9) | 0.977 |
| Purposefully selected villages | 28.0 (24.8) | 31.4 (22.4) | 24.2 (17.7) | 0.855 |
| Randomly selected villages | 5.7 (8.3) | 2.2 (3.0) | 5.4 (5.7) | 0.380 |
| Number of annual canine Guinea worm cases per village, 2012–2019, mean (SD) ^†^ |  |  |  |  |
| Overall | 16.5 (20.9) | 17.9 (23.5) | 21.3 (24.5) | 0.842 |
| Purposefully selected villages | 38.3 (28.7) | 45.0 (21.9) | 36.8 (31.0) | 0.880 |
| Randomly selected villages | 8.6 (10.5) | 4.4 (5.9) | 11.0 (12.3) | 0.344 |
| Years under active surveillance per village, mean (SD) ^†^ | 4.5 (1.9) | 5.6 (1.4) | 4.9 (1.8) | 0.224 |
| Female, *n* (%) | 76 (49.7) | 85 (53.1) | 90 (58.1) | 0.332 |
| Age in years, mean (SD) ^†^ | 37 (15.0) | 34 (14.8) | 37 (15.1) | 0.280 |
| Completion of primary school or higher,  *n* (%) | 24 (15.7) | 31 (19.4) | 26 (16.8) | 0.674 |
| Farmer, *n* (%) | 121 (79.1) | 115 (71.9) | 109 (70.3) | 0.176 |
| Dog owners, *n* (%) | 50 (32.7) | 51 (31.9) | 85 (54.8) | **<.0001** |
| Cat owners, *n* (%) | 41 (26.8) | 43 (26.9) | 49 (31.6) | 0.549 |
| Visited by a volunteer at least twice per week, *n* (%) | 69 (45.1) | 89 (55.6) | 90 (58.1) | 0.053 |
| Never visited by a volunteer, *n* (%) | 30 (19.6) | 18 (11.3) | 27 (17.4) | 0.111 |
| Can identify photo of Guinea worm, *n* (%) | 146 (95.4) | 154 (96.3) | 152 (98.1) | 0.758 |
| Sources of Guinea worm information^¶^ |  |  |  |  |
| Village volunteer, *n* (%) | 104 (68.0) | 100 (62.5) | 104 (67.1) | 0.546 |
| Health facility, *n* (%) | 14 (9.2) | 26 (16.3) | 14 (9.0) | 0.071 |
| CGWEP staff *, *n* (%) | 61 (39.9) | 111 (69.4) | 127 (81.9) | **<.0001** |
| Village chief, *n* (%) | 0 (0.0) | 2 (1.3) | 3 (1.9) | 0.246 |
| Teacher/school, *n* (%) | 1 (0.7) | 2 (1.3) | 2 (1.3) | 0.831 |
| Radio/television, *n* (%) | 5 (3.3) | 6 (3.8) | 10 (6.5) | 0.345 |
| Theater, *n* (%) | 0 (0.0) | 0 (0.0) | 0 (0.0) | --- |
| Mosque/church, *n* (%) | 0 (0.0) | 0 (0.0) | 1 (0.7) | 0.364 |
| Market day, *n* (%) | 0 (0.0) | 3 (1.9) | 3 (1.9) | 0.228 |
| Town crier, *n* (%) | 0 (0.0) | 1 (0.6) | 2 (1.3) | 0.365 |
| Poster, *n* (%) | 1 (0.7) | 3 (1.9) | 4 (2.6) | 0.419 |
| Guinea worm symptoms named^¶^ |  |  |  |  |
| Itching, *n* (%) | 64 (41.8) | 91 (56.98) | 91 (58.7) | **0.005** |
| Burning, *n* (%) | 12 (7.8) | 25 (15.6) | 26 (16.8) | **0.044** |
| Pain, *n* (%) | 7 (4.6) | 36 (22.5) | 19 (12.3) | **<.0001** |
| Swelling, *n* (%) | 53 (34.6) | 52 (32.5) | 70 (45.2) | **0.0467** |
| Blister, *n* (%) | 43 (28.1) | 58 (36.3) | 87 (56.1) | **<.0001** |
| Wound, *n* (%) | 53 (34.6) | 95 (59.4) | 82 (52.9) | **<.0001** |
| Emerging worm, *n* (%) | 47 (30.7) | 59 (36.9) | 43 (27.7) | 0.206 |
| Total number of symptoms identified, mean (SD) | 1.82 (1.4) | 2.60 (1.7) | 2.70 (1.6) | **<.0001** |
| Zero symptoms named, *n* (%) | 34 (22.2) | 21 (13.1) | 26 (16.8) | 0.102 |
| Reasons named for reporting Guinea worm^¶^ |  |  |  |  |
| To receive a reward, *n* (%) | 10 (6.5) | 60 (37.5) | 30 (19.4) | **<.0001** |
| To get care, *n* (%) | 49 (32.0) | 113 (70.6) | 75 (48.4) | **<.0001** |
| To protect the community, *n* (%) | 12 (7.8) | 1 (0.6) | 20 (12.9) | **0.000** |
| For the health of the community,  *n* (%) | 15 (9.8) | 10 (6.3) | 18 (11.6) | 0.245 |
| To stop the transmission of Guinea worm, *n* (%) | 11 (7.2) | 8 (5.0) | 19 (12.3) | 0.054 |
| To eradicate Guinea worm, *n* (%) | 1 (0.7) | 7 (4.4) | 12 (7.7) | **0.009** |
| Total number of reasons for reporting identified, mean (SD) | 0.64 (0.6) | 1.24 (0.7) | 1.12 (0.7) | **<.0001** |
| Zero reasons named, *n* (%) | 68 (44.4) | 24 (15.0) | 19 (12.3) | **<.0001** |
| Reward system, knowledge of ____: |  |  |  |  |
| The reward for reporting Guinea worm in a person, *n* (%) | 112 (73.2) | 142 (88.8) | 145 (93.6) | **<.0001** |
| Amount for self-reporting Guinea worm in yourself, *n* (%) | 54 (35.3) | 68 (42.5) | 87 (56.1) | **0.001** |
| Amount if someone other than the person with Guinea worm reports Guinea worm, *n* (%) | 40 (26.1) | 58 (36.3) | 84 (54.2) | **<.0001** |
| The reward for reporting Guinea worm in dogs or cats, *n* (%) | 127 (83.0) | 127 (79.4) | 144 (92.9) | **0.000** |
| The requirement dogs or cats with Guinea worm must be leashed until the wounds are fully healed to earn an animal reward, *n* (%) | 61 (39.9) | 110 (68.8) | 97 (62.6) | **<.0001** |
| Amount if the owner of a dog or cat reports that their animal has a blister or swelling *before* a Guinea worm emerges, *n* (%) | 58 (37.9) | 47 (29.4) | 88 (56.8) | **<.0001** |
| Amount if someone other than the owner of the dog or cat reports an animal with a blister or swelling *before* a Guinea worm emerges, *n* (%) | 30 (19.6) | 33 (20.6) | 66 (42.6) | **<.0001** |
| Amount if the owner of the dog or cat reports an animal with a wound or an emerging Guinea worm, *n* (%) | 16 (10.5) | 8 (5.0) | 26 (16.8) | **0.003** |
| Amount if someone other than the owner of the dog or cat reports an animal with a wound or an emerging Guinea worm, *n* (%) | 12 (7.8) | 5 (3.1) | 21 (13.6) | **0.003** |
| Amount if owner of a dog or cat reports any signs and symptoms of Guinea worm and no worm emerges, *n* (%) | 6 (3.9) | 14 (8.8) | 44 (28.4) | **<.0001** |
| Amount if someone other than the owner of the dog or cat reports any signs and symptoms of Guinea worm and no worm emerges, *n* (%) | 24 (15.7) | 28 (17.5) | 46 (29.7) | **0.004** |
| Guinea worm prevention strategies named^¶^ |  |  |  |  |
| Drinking safe water, *n* (%) | 39 (25.5) | 50 (31.3) | 38 (24.5) | 0.347 |
| Filtering unsafe water, *n* (%) | 44 (28.8) | 45 (28.1) | 34 (21.9) | 0.320 |
| Preventing patients from entering water sources, n (%) | 4 (2.6) | 5 (3.1) | 8 (5.2) | 0.448 |
| Proper disposal of fish entrails, *n* (%) | 59 (38.6) | 94 (58.8) | 84 (54.2) | **0.001** |
| Proper cooking of fish and aquatic animals, *n* (%) | 25 (16.3) | 68 (42.5) | 85 (54.8) | **<.0001** |
| Tethering infected dogs/cats to prevent them from entering water sources,  *n* (%) | 3 (2.0) | 9 (5.6) | 16 (10.3) | **0.008** |
| Total number of prevention strategies identified, mean (SD) | 1.1 (1.2) | 1.69 (1.1) | 1.71 (1.2) | **<.0001** |
| Zero prevention strategies named, *n* (%) | 61 (39.9) | 30 (18.8) | 32 (20.7) | **<.0001** |

* Fifteen villages were selected in each of the three geographic areas, including 15 from the Bailli area (recent increase in canine cases), 15 villages from the Mandelia area (moderate incidence of canine cases), and 15 from the Sarh area (high incidence of canine cases).

^†^ SD=standard deviation.

^¶^ These questions were open-ended and multiple answers were permitted.

**S3 Table: Villager-Level Models (Supplement): The impact of years of active surveillance on Guinea worm knowledge and volunteer visit frequency (n=468 villagers) in the survey to evaluate the Chad Guinea Worm Eradication Program active surveillance system: September 2019.** Guinea worm knowledge was assessed by symptoms named, strategies named, and reasons for reporting Guinea worm.

|  | **Outcomes** | | | | | | | |
| --- | --- | --- | --- | --- | --- | --- | --- | --- |
|  | > 2 GW symptoms named^⍏^ | | > 2 GW prevention  strategies named^¶^ | | Any reasons for reporting GW named^§^ | | Visited by a volunteer  > 2 times per week | |
| **Predictor Variable** | OR (95% CI) | AIC | OR (95% CI) | AIC | OR (95% CI) | AIC | OR (95% CI) | AIC |
|  |  |  |  |  |  |  |  |  |
| >5 years of active surveillance in the village* | **0.39 (0.23-0.65)** | 551.90 | 0.69 (0.44-1.11) | 648.46 | 0.76 (0.38-1.51) | 510.15 | **0.55 (0.35-0.87)** | 642.83 |

Bold indicates statistical significance (p<0.05).

*Adjusted for clustering by village and number of dogs with Guinea worm at the village-level, 2018–2019.

^⍏^Symptoms include itching, burning, pain, swelling, blister, wound, and emerging worm.

^¶^Prevention strategies include drinking safe water, filtering unsafe water, preventing patients from entering water sources, proper disposal of fish entrails, proper cooking of fish and aquatic animals, and tethering infected dogs/cats to prevent them from entering water sources.

^§^Reasons for reporting include to receive a reward, to get care, to protect the community, for the health of the community, to stop the transmission of Guinea worm, and to eradicate Guinea worm.
