# Supplementary material for: Community-based Guinea worm surveillance in Chad: Evaluating a system at the intersection of human and animal disease
Source: PLoS Negl Trop Dis. 2021 Mar 18;15(3):e0009285. doi: 10.1371/journal.pntd.0009285 (PMC8023463; doi:10.1371/journal.pntd.0009285)
Supplement: S3 Table — (DOCX) [file pntd.0009285.s003.docx]

**S3 Table: Villager-Level Models (Supplement): The impact of years of active surveillance on Guinea worm knowledge and volunteer visit frequency (n=468 villagers) in the survey to evaluate the Chad Guinea Worm Eradication Program active surveillance system: September 2019.** Guinea worm knowledge was assessed by symptoms named, strategies named, and reasons for reporting Guinea worm.

|  | **Outcomes** | | | | | | | |
| --- | --- | --- | --- | --- | --- | --- | --- | --- |
|  | > 2 GW symptoms named^⍏^ | | > 2 GW prevention  strategies named^¶^ | | Any reasons for reporting GW named^§^ | | Visited by a volunteer  > 2 times per week | |
| **Predictor Variable** | OR (95% CI) | AIC | OR (95% CI) | AIC | OR (95% CI) | AIC | OR (95% CI) | AIC |
|  |  |  |  |  |  |  |  |  |
| >5 years of active surveillance in the village* | **0.39 (0.23-0.65)** | 551.90 | 0.69 (0.44-1.11) | 648.46 | 0.76 (0.38-1.51) | 510.15 | **0.55 (0.35-0.87)** | 642.83 |

Bold indicates statistical significance (p<0.05).

*Adjusted for clustering by village and number of dogs with Guinea worm at the village-level, 2018–2019.

^⍏^Symptoms include itching, burning, pain, swelling, blister, wound, and emerging worm.

^¶^Prevention strategies include drinking safe water, filtering unsafe water, preventing patients from entering water sources, proper disposal of fish entrails, proper cooking of fish and aquatic animals, and tethering infected dogs/cats to prevent them from entering water sources.

^§^Reasons for reporting include to receive a reward, to get care, to protect the community, for the health of the community, to stop the transmission of Guinea worm, and to eradicate Guinea worm.
